# Effects of a Physiotherapist-Led School-Based Health Education Workshop on Spinal Pain Prevention in Schoolchildren: A Quasi-Experimental Study

**DOI:** 10.3390/healthcare14111525

**Published:** 2026-05-30

**Authors:** Manuel Fraiz-Barbeito, Sara Rey-Veiga, María Teresa Santamaría-Solís, Yoana González-González, Mercedes Soto-González, Iria Da Cuña-Carrera, Alejandra Alonso-Calvete

**Affiliations:** 1 Servicio de Rehabilitación, Complexo Hospitalario Universitario de Pontevedra (CHOP), Sergas, 36071 Pontevedra, Spain; manuel.fraiz.barbeito@sergas.es (M.F.-B.); sara.rey.veiga@sergas.es (S.R.-V.); maria.teresa.santamaria.solis@sergas.es (M.T.S.-S.); 2 Departamento de Biología Funcional y Ciencias de la Salud, Facultad de Fisioterapia, Universidad de Vigo, 36005 Pontevedra, Spain; m.soto@uvigo.gal (M.S.-G.); iriadc@uvigo.gal (I.D.C.-C.); alejalonso@uvigo.gal (A.A.-C.); 3 Grupo de Investigación Fisioterapia Clínica (FS1), Instituto de Investigación Sanitaria Galicia Sur (IIS Galicia Sur), SERGAS-UVIGO, 36005 Pontevedra, Spain; 4 Grupo de Investigación REMOSS, Equipo de Investigación en Rendimiento y Motricidad del Salvamento y Socorrismo, 36005 Pontevedra, Spain

**Keywords:** back pain, neck pain, spinal diseases, child, schools, ergonomics, health education, prevention, health promotion, physical therapy modalities

## Abstract

**Highlights:**

**What are the main findings?**

- The study shows that a physiotherapist-led, school-based theoretical–practical program improves children’s knowledge about spinal health and ergonomic risk factors associated with spinal pain.
- The intervention enhances children’s ability to identify healthy habits during everyday activities, addressing the cervical, thoracic, and lumbar regions in an integrated manner.

**What are the implications of the main findings?**

- The results support the role of the school as a key setting for secondary prevention of spinal pain, especially considering children’s increasing exposure to prolonged postures, sedentary behavior, and intensive use of electronic devices.
- Second bullet. The evidence reinforces the need to implement standardized, active, and multidimensional spinal health education programs capable of reducing the future burden of chronic pain and promoting healthy lifestyle habits from an early age.

**Abstract:**

**Background/Objectives:** Spinal pain is common among schoolchildren and is associated with poor postural habits and sedentary behavior. Schools represent an optimal setting for prevention; however, they are also key environments for prevention strategies in children who already experience spinal pain. This study focused on children aged 9–11 years and aimed to evaluate the effectiveness of a physiotherapist-led, classroom-based workshop as a prevention intervention to improve spinal pain outcomes. **Methods:** A quasi-experimental single-group pre–post intervention study was conducted in public primary schools. The intervention consisted of two 45 min theoretical–practical sessions. A 21-item questionnaire assessed spinal pain, postural habits, backpack-related behaviors, physical activity, screen use and spinal literacy at baseline and three months post-intervention. McNemar and Wilcoxon tests were applied (*p* < 0.05). **Results:** A total of 287 schoolchildren participated. Cervical and thoracic pain decreased significantly (*p* = 0.036; *p* = 0.040), while lumbar pain showed no change. Postural habits improved: sitting with back support increased (+12.7%; *p* < 0.001), sitting on the chair edge decreased (−10.5%; *p* < 0.001), and side-lying sleeping increased (*p* = 0.006). Knowledge of proper backpack load distribution also improved (+17.9%; *p* < 0.001). No significant changes were observed in physical activity, screen use, rising-from-bed technique, or backpack type. **Conclusions:** The workshop improved upper-spine pain, spinal literacy and modifiable habits, while automated motor behaviors and family-dependent routines showed limited change.

## 1. Introduction

Spinal pain, which encompasses the cervical, thoracic, and lumbar regions, has become a major public health problem that increasingly affects pediatric and adolescent populations [1]. Current estimates place the prevalence of this type of pain during adolescence between 7% and 70% [2], with an average of 39.9% [3], reaching levels comparable to those observed in adulthood by the end of the school years [4]. The clinical relevance of this phenomenon is critical, as experiencing back pain during childhood is a significant predictor of chronic pain and disability in adulthood [5,6].

The etiology of these conditions is multifactorial [7] and should be examined from the perspective of the biopsychosocial conceptual model, as back pain arises from a complex interaction among biological factors (such as genetics and growth), psychological factors (such as anxiety or stress), and social or environmental factors [8,9]. During the school day, students are exposed to persistent school-related ergonomic risks, such as maintaining prolonged static postures for up to 92% of classroom time, the use of inadequate furniture that does not match their anthropometric development [10,11], and carrying excessively heavy backpacks [12], which should not exceed 10–15% of the child’s body weight to avoid overload. These factors are further aggravated by modern lifestyle patterns characterized by increasing sedentarism and intensive use of electronic devices, which promote incorrect postures [13,14], exacerbating musculoskeletal strain and alterations in the sagittal curvature of the spine [15].

Given that school is the environment where children spend most of their active daily time, it is considered the ideal setting for developing effective health education programs aimed at primary prevention and the promotion of spinal health [16]. Schools are a vital resource for influencing students’ well-being [11]; school-based education is the most realistic and feasible approach [17] and also the one that allows reaching the largest number of individuals proactively [18]. The effectiveness of these back-health education programs (“Back Schools”) in improving knowledge (literacy) and postural behavior is supported by the review of [12], as school-based interventions enhance personal skills and empowerment through increased health literacy [19] and improve knowledge and positive attitudes toward spinal care [2].

Scientific evidence suggests the superiority of interventions that combine theory, practice, and exercise over purely theoretical models [4,19]. These authors recommend educational approaches that integrate theoretical instruction on postural hygiene with ergonomic practice, postural training, and therapeutic strengthening and flexibility exercises [20].

Despite the recognition of these benefits, the scientific literature still shows a notable lack of consensus regarding specific teaching guidelines and a high heterogeneity in the evaluation instruments used, reflecting the absence of standardized tools (questionnaires) and the wide variety of programs applied [21]. A major limitation frequently highlighted is the presence of inconsistent outcome measures across studies [12]. Furthermore, most research has traditionally focused exclusively on low back pain, often neglecting other spinal regions such as the cervical and thoracic spine. Systematic reviews and epidemiological studies have highlighted this limitation, showing that the majority of interventions and outcome measures in school-based populations are centered on lumbar pain alone [4,12,22]. This narrow focus limits a comprehensive understanding of spinal health, despite evidence that pain in different spinal regions frequently coexists and may share common risk factors. Therefore, a global approach addressing the cervical, thoracic, and lumbar spine is warranted. Therefore, it is essential to conduct rigorous epidemiological studies that evaluate the effects of health education programs addressing neck, thoracic, and lumbar pain jointly [23], to further investigate environmental and personal factors that guide schoolchildren toward appropriate management strategies [13], and to update prevalence data to inform and improve prevention strategies [1].

The aim of this study was to evaluate the effectiveness of a physiotherapist-led, classroom-based health education workshop in improving schoolchildren’s knowledge of spinal health and promoting preventive habits to reduce the risk of spinal pain.

## 2. Materials and Methods

### 2.1. Study Design

A quasi-experimental single-group pre–post intervention multicenter study was conducted in public primary schools in the municipality of Pontevedra, including the entire period from the 2018–2019 to the 2022–2023 school years. The intervention consisted of a physiotherapist-led health education program delivered as a theoretical-practical classroom-based workshop. The study included two assessment points: baseline and three months.

### 2.2. Participants

Students from the 4th and 5th grade of Primary Education, aged 9 to 11 years, were included in the study. Inclusion required approval from the school administration and the Parents’ Association, as well as the commitment of the teaching staff to administer the questionnaires on the scheduled dates. Schools that declined participation were excluded, as well as students with cognitive difficulties that could prevent them from adequately completing the questionnaires.

### 2.3. Sample Size Calculation

The sample size was calculated using the 34.3% prevalence of spinal pain reported by Fraiz-Barbeito et al. (2021) [1] in Spanish schoolchildren aged 9–11 years. Assuming a 95% confidence level (Z = 1.96) and a precision of 4.7%, the minimum required sample size was estimated to be 300 participants. The sample size was initially estimated using the reported prevalence of spinal pain (34.3%) in Spanish schoolchildren to ensure an adequate number of eligible participants in a real-world school-based recruitment context. As participant inclusion was based on classroom-level screening rather than pre-selected symptomatic individuals, this approach allowed us to account for the expected proportion of students with spinal pain. However, given that the final sample included only participants with existing pain, a sample size calculation based on expected effect size would have been methodologically more appropriate. This is acknowledged as a limitation of the study.

### 2.4. Intervention

The intervention consisted of a theoretical–practical workshop delivered by physiotherapists, structured into two 45 min sessions held 2–3 weeks apart. The sessions took place in the students’ regular classrooms during school hours, within the subject Environmental Knowledge.

The workshop content included a theoretical component covering fundamental concepts of spinal anatomy and biomechanics, as well as a practical component in which demonstrations and hands-on practice of postural hygiene principles were carried out. These principles were applied to everyday activities such as sitting, standing up, lying down, walking, and handling school backpacks. Additionally, the program addressed the importance of regular extracurricular physical activity and provided recommendations on the appropriate use of electronic devices as a strategy to reduce sedentary behavior and promote healthy habits.

To reinforce the learning process, students received an informational leaflet summarizing the key messages of the program, and each participating school displayed an A2 poster highlighting the main recommendations for spinal health.

The duration and timing of the intervention were defined based on both practical and pedagogical considerations. Each session lasted 45 min to match standard school class periods and ensure feasibility within the regular curriculum. The 2–3 week interval between sessions was designed to facilitate consolidation of the concepts introduced in the first session, allowing students to apply the recommendations in their daily routines before receiving reinforcement. This approach is consistent with previous school-based educational interventions and with learning principles that support spaced repetition as a strategy to enhance knowledge retention and behavioral change. Additionally, this structure was informed by prior experience and pilot implementations conducted by the research team in similar school contexts.

### 2.5. Measurement Instruments

A 21-item ad hoc multiple-choice questionnaire, developed based on previously validated instruments, was used to assess the following variables: presence of spinal pain (cervical, thoracic, and lumbar regions), pain duration (less than 12 h, 12–24 h, 1–7 days, or more than one week), and pain intensity using the Wong–Baker FACES scale. The questionnaire also evaluated postural habits (sitting posture, sleeping posture, and method of rising from bed), backpack-related behaviors (including design characteristics, weight distribution, organization, inspection routines, and carrying method), and lifestyle factors such as physical activity and screen use (measured by weekly frequency and daily duration).

The instrument was developed based on previously validated tools assessing spinal pain and postural habits in schoolchildren [24,25]. It underwent expert review and pilot testing to ensure clarity and content validity. However, formal psychometric validation (e.g., test–retest reliability or internal consistency) was not conducted and should be addressed in future research. All questionnaires were anonymous and self-administered.

### 2.6. Procedure

Teachers administered the baseline questionnaire before the start of the workshop and again three months after the intervention. The research team subsequently collected all completed questionnaires for analysis. The intervention spanned the entire period from the 2018–2019 to the 2022–2023 school years.

### 2.7. Ethical Considerations

The study received approval from the Research Ethics Committee of Galicia and authorization from the Pontevedra-Salnés Integrated Management Directorate. All procedures complied with current Spanish regulations and the principles of the Declaration of Helsinki. Participant anonymity was ensured, and no identifiable data were collected.

### 2.8. Statistical Analysis

In the pre–post analysis, the effects of the intervention were evaluated using statistical tests for related data. The normality of quantitative variables was assessed using the Kolmogorov–Smirnov test; as the assumption of normality was not met, non-parametric tests were applied. Dichotomous variables were analyzed using McNemar’s test, while ordinal and non-parametric quantitative variables were compared using the Wilcoxon signed-rank test. For categorical variables with more than two categories, recategorized comparisons were performed to identify specific patterns of change. The significance level was set at *p* < 0.05. Effect size (ES) was calculated using Cohen’s *h*. An ES < 0.20 is low, <0.5 is medium and from 0.8 is high.

### 2.9. Data Availability and Use of Generative Artificial Intelligence

All generated data are anonymous and presented in aggregated form; no individual-level datasets were produced that would require deposition in public repositories.

No generative artificial intelligence was used in the study design, data collection, or statistical analysis; it was employed exclusively for minor linguistic editing tasks.

## 3. Results

A total of 317 schoolchildren aged 9 to 11 years were initially assessed (51.2% girls; mean weight: 35.46 ± 0.51 kg; 89.4% right-handed). After excluding incomplete questionnaires, the final analytical sample comprised 287 participants. All students completed the assessment at baseline and again three months after the intervention. Table 1 presents the descriptive characteristics of the sample.

The following sections present the pre- to post-intervention results for each measurement domain included in the 21-item questionnaire: spinal pain variables, postural habits, backpack-related behaviors, physical activity, screen use, and spinal literacy.

### 3.1. Changes in Spinal Pain

Regarding the distribution of spinal pain, cervical pain showed the highest baseline prevalence (22.3%, n = 64), followed by thoracic pain (17.4%, n = 50) and lumbar pain (5.9%, n = 17). After the intervention, an overall reduction was observed: cervical pain decreased to 14.6% (n = 42; *p* = 0.036; ES = 0.20, moderate), thoracic pain to 12.8% (n = 35; *p* = 0.040; ES = 0.13, low), and lumbar pain to 4.1% (n = 12; *p* = 0.458). No significant differences were found between sexes (*p* > 0.05), nor were changes observed in pain duration (*p* = 0.747) or in intensity as measured by the Wong–Baker FACES scale (*p* = 0.081).

Overall, the cervical and thoracic regions experienced the most notable and statistically significant improvements.

Regarding pain intensity, baseline pain intensity was moderate (mean ≈ 4.13; median = 5). Post-intervention, a slight decrease in mean values was observed (≈3.69), while the median remained unchanged (5), indicating minimal distributional shift in pain intensity across participants.

### 3.2. Improvements in Postural Habits

Sleeping posture.

A significant improvement was observed: prone sleeping decreased by 12% (*p* < 0.009; ES = 0.22, moderate), while lateral sleeping increased from 71% to 79.5% (*p* = 0.006; ES = 0.20, moderate).

Method of rising from bed.

No significant change was detected in the technique used to get out of bed (*p* = 0.170).

Sitting posture.

Improvements were notable. Sitting at the edge of the chair decreased by 10.5% (*p* < 0.001; ES = 0.49, moderate), while sitting with the back supported increased by 12.7% (*p* < 0.001; ES = 0.19, low).

### 3.3. Improvements in Backpack-Related Behaviors

Significant improvements were recorded in various aspects of backpack management. The proportion of participants placing heavier books closest to the back increased by 17.9% (*p* < 0.001; ES = 0.4, moderate), while those considering weight distribution “irrelevant” decreased by 8.6% (*p* < 0.001).

Additionally, identifying the correct method for carrying the backpack while ascending stairs (centered on the back with both straps) increased by 10% (*p* = 0.002; ES = 0.25, moderate), while the belief that ascending stairs while holding the backpack by the top handle was correct decreased by 5.7% (*p* = 0.04; ES = 0.21, moderate).

No significant changes were observed in other variables, such as the type of backpack (*p* = 0.839), general method of placing books (*p* = 0.092), positioning the backpack on the back (*p* = 0.285), or the timing of backpack inspection (*p* = 0.534).

### 3.4. Changes in Physical Activity

No statistically significant changes were observed in the general practice of extracurricular sports following the intervention (*p* > 0.05). However, a significant reduction was identified in the number of days per week that participants engaged in physical activity. Median weekly frequency decreased from approximately 2–3 days at baseline to around 1 day post-intervention (*p* < 0.001). Despite this reduction in frequency, no significant differences were found in the number of hours of physical activity performed per day (*p* > 0.05).

### 3.5. Changes in Screen Use

No statistically significant changes were detected in electronic device use following the intervention. The number of days per week in which participants reported using electronic devices remained stable (*p* > 0.05). Daily duration of device use also showed no meaningful change between baseline and follow-up assessments (*p* > 0.05).

### 3.6. Improvements in Spinal Literacy

Knowledge regarding healthy postural habits improved significantly. The proportion of students who believed prone sleeping was appropriate decreased by 4.2% (*p* = 0.012). Furthermore, recognition of the correct technique for rising from bed—rolling to one side, sitting up, and then standing—increased by 12.5% (*p* < 0.001).

## 4. Discussion

The present study examined the effects of a school-based health education intervention aimed at preventing and managing spinal pain in children aged 9 to 11 years. The findings suggest that the program may contribute to reductions in cervical and thoracic pain, as well as to improvements in certain postural habits related to sitting, sleeping, and backpack use and to increased health literacy regarding spinal care. In contrast, no significant changes were observed in lumbar pain or in more automatic motor behaviors—such as getting out of bed or carrying the backpack on stairs—which may indicate that these patterns are less responsive to classroom-based education alone and could require more intensive, practical, or supervised training. Overall, the results appear to indicate that the intervention was more effective in influencing high-exposure behaviors and postural beliefs, while having a more limited impact on deeply ingrained motor habits or those influenced by family-related routines. The following sections explore these findings in greater detail, focusing on changes in pain distribution, postural habits, and load-management behaviors.

### 4.1. Changes in Cervical, Thoracic, and Lumbar Pain

Following the intervention, cervical and thoracic pain decreased significantly, whereas lumbar pain showed no meaningful variation. The improvements observed in the cervical and thoracic regions align with previous work indicating that upper spine pain is more prevalent in school-aged children and responds more rapidly to changes in school-related habits, largely due to their sustained exposure to reading and writing postures, intensive use of electronic devices, and daily physical activity [1]. Within this context, the intervention’s focus on postural hygiene, healthy physical activity, and regulated screen use likely exerted a direct influence on these high-exposure behaviors, contributing to the reduction in cervical and thoracic pain.

In contrast, the absence of significant changes in lumbar pain may be explained by several factors. First, the lower baseline prevalence of lumbar pain in our sample may have limited the statistical power to detect significant differences. Second, lumbar pain in children is often more closely associated with physical conditioning, motor control, and habitual movement patterns, which typically require longer or more intensive interventions to achieve meaningful improvement. Furthermore, the intervention primarily targeted postural and school-related ergonomic behaviors, which are more directly linked to cervical and thoracic loading, whereas lumbar-related behaviors may require greater motor automatization and sustained practice. These differences may explain the distinct response observed across spinal regions.

These patterns also help contextualize why no differences were observed according to sex, pain duration, or baseline intensity, as explored in the next section.

### 4.2. Absence of Differences by Sex, Pain Intensity, and Pain Duration

No significant differences were observed according to sex, pain intensity, or pain duration. This finding is consistent with the meta-analysis by [26], which reported that musculoskeletal pain—including spinal pain—is the only type of childhood pain that does not show sex-related differences prior to adolescence.

Baseline pain intensity was moderate (mean ≈ 4.13; median = 5), indicating that a substantial proportion of participants already presented low to moderate pain levels at the start of the study. Following the intervention, pain intensity showed only a slight reduction (mean ≈ 3.69; median = 5), with the median remaining unchanged. This limited shift suggests that many participants remained within similar pain categories after the intervention. Together, these findings support the presence of a potential floor effect, which may have restricted the capacity to detect statistically significant improvements in pain intensity. Therefore, the lack of pronounced changes should be interpreted cautiously and in light of the relatively low baseline values.

Although López Hernández et al. [27] do not explicitly analyze this phenomenon, their data indicate that a proportion of schoolchildren report mild pain that is not associated with external factors such as backpack weight, suggesting low clinical variability and, consequently, reduced capacity for change in response to short-duration educational interventions.

### 4.3. Changes in Postural Habits: Impact on Sitting and Sleeping

One of the strengths of the intervention appears to have been the improvement observed in habits related to sedentary behavior, as a 10.5% decrease was recorded in the proportion of students sitting on the edge of the chair, along with a 12.7% increase in correct back support. This finding is particularly relevant given that children spend up to 92% of the school day in a seated position, as reported by Alibegović et al. (2020) [10], and prolonged static sitting has been associated with increased thoracic kyphosis and alterations in lumbar lordosis. Sustained exposure to inadequate furniture and habitual sitting during academic tasks contributes to dysfunctional postural patterns, increased mechanical load on the paravertebral musculature, and a higher risk of musculoskeletal pain. These observations are consistent with prior reviews highlighting that improving classroom ergonomics and reinforcing postural hygiene can mitigate these risks and promote healthier sitting behaviors.

In line with this evidence, Ozdemir (2021) [22] documented that up to 86% of adolescents adopt inadequate sitting postures in the classroom, a pattern linked to postural imbalance and greater risk of musculoskeletal symptoms, as also noted by Li et al. (2022) [15]. Additionally, several studies have shown that incorrect sitting postures substantially increase the likelihood of low back pain in school-aged populations, with elevated risk associations reported by Minghelli et al. (2021) [19]. The improvements reported in our study align with findings from García-Moreno et al. (2024) [4] and Galmes-Panadés et al. (2023) [7], who identify sitting behavior as one of the most modifiable habits in the school context due to children’s high daily exposure and the continuous corrective feedback provided by teachers, which facilitates immediate correction and consolidation of healthier postural patterns.

With respect to sleeping posture, the reduction in prone sleeping (from 12.3% to 6%) and the increase in lateral sleeping (up to 79.5%) indicate a clear enhancement in postural literacy. This change is important because sleep posture influences pediatric lumbar pain; Fraiz-Barbeito et al. (2021) [1] found that children experiencing spinal pain tend to avoid the prone position as a protective strategy, supporting the shift toward lateral decubitus as an effective protective behavior. Although sleep-related habits are difficult to modify due to their automatic nature [5], our findings suggest that combining theoretical instruction with practical components is more effective than purely informational interventions.

In contrast, no significant changes were observed in the way students got out of bed or carried their backpacks on stairs. These behaviors require motor automatization and repeated practice, making them less responsive to short-duration educational interventions. Akbari-Chehrehbargh et al. (2020) [28] and Minghelli et al. (2021) [19] note that cognitive improvements typically precede behavioral changes, a pattern reflected in the present study: knowledge and beliefs improved, but actual motor behavior did not, likely due to limited body awareness and insufficient proprioceptive training.

The literature indicates that the effectiveness of Back Schools depends on incorporating practical and individualized strategies, as theoretical learning alone does not ensure stable behavioral change. While certain habits—such as sitting posture—can be corrected through continuous feedback from teachers, other private or less observable behaviors (e.g., getting out of bed) lack direct supervision and require systematic reinforcement at home by parents and caregivers, who act as natural monitors of everyday routines. In this regard, the meta-analysis by Anyachukwu et al. (2024) [12] demonstrates that behaviors dependent on the home environment or motor repetition show limited capacity for change unless school-based interventions are combined with family support and guided practice. However, not all postural behaviors demonstrated the same degree of modifiability, particularly those influenced by household routines and equipment availability, as discussed below.

### 4.4. Limited Changes in Backpack-Related Behaviors

No significant changes were observed in variables related to backpack use, such as backpack type or the time of day students checked their backpacks. These findings could indicate that such behaviors depend on other factors, such as family decisions. As noted by Anyachukwu et al. (2024) [12], postural education improves knowledge but does not necessarily modify habits rooted in the household environment, particularly those linked to school equipment, which require financial investment and stable parental decision-making. Likewise, research in school ergonomics indicates that the persistent use of inadequate backpacks often reflects socioeconomic constraints that act as external barriers to the effectiveness of educational interventions, limiting the modifiability of behaviors shaped by family context. Despite these behavioral limitations, the intervention produced meaningful improvements in health literacy, particularly regarding load-management strategies, which are discussed in the following section.

### 4.5. Improvements in Backpack-Related Behavior Literacy

The intervention produced a significant improvement in students’ knowledge regarding appropriate backpack load distribution (+17.9%; *p* < 0.001), a finding consistent with studies showing that postural education enhances schoolchildren’s understanding of load-handling principles. Similar gains in ergonomic knowledge have been reported in brief school-based interventions such as those by Pani et al. (2024) [11], while Akbari-Chehrehbargh et al. (2020) [28] documented a 36.4% increase in self-care knowledge following a structured educational program. Likewise, the work of López-Hernández et al. (2020) [27] highlights the need to better understand how schoolchildren manage their backpacks and notes the absence of an association between backpack weight and musculoskeletal pain, underscoring the value of educational strategies that strengthen awareness and postural management beyond simply controlling carried load.

The belief that the backpack should be centered when climbing stairs also increased (+10%; *p* = 0.002), reflecting a cognitive improvement that did not translate into behavioral change (*p* = 0.285). This pattern mirrors observations by Akbari-Chehrehbargh et al. (2020) [28], who found that educational interventions tend to improve knowledge and beliefs, whereas behavioral change requires more time and practice. Similarly, the meta-analysis by Anyachukwu et al. (2024) [12] indicates that school-based back-care programs do not consistently modify complex motor behaviors or those influenced by the home environment. Taken together, these findings suggest that knowing what to do does not guarantee the automatization of motor habits, which require repetition, supervision, and practical reinforcement beyond the classroom.

### 4.6. Physical Activity Findings

No improvements were observed in sports participation or daily exercise duration, and weekly frequency significantly decreased despite the workshop’s emphasis on promoting regular physical activity. This decline aligns with the pandemic context, a period during which children’s physical activity levels dropped markedly [29] due to restrictions that limited group and sports activities and hindered any potential effect of the intervention.

It should also be noted that the study did not collect detailed information on the type or context of physical activity (e.g., structured sports vs. unstructured play), which limits the interpretation of these results. Different activity types may follow distinct participation patterns and could partially explain this discrepancy.

Additionally, differences in how children perceive and report frequency versus duration may have contributed to this result. These findings should therefore be interpreted with caution, and future studies should incorporate more detailed measures of physical activity to better understand these patterns.

### 4.7. Screen Use Findings

No clear changes were observed in electronic device use. This result may be consistent with previous evidence suggesting that screen-related habits can be difficult to modify and may be influenced by factors such as family routines and access to technology. The results are consistent with those of Basuodan et al. (2024) [30], who reported an increase in screen time during and after the lockdown and highlighted that this behavior is closely tied to the family environment and household access to digital devices, thereby limiting the impact of school-based interventions alone. Furthermore, Basuodan et al. (2024) [30] demonstrated an inverse relationship between physical activity and screen time, indicating that greater device use is associated with lower physical activity levels. This reinforces the notion that established digital habits within the home act as external barriers that hinder both increasing physical activity and reducing screen exposure, even when educational messages are reinforced at school.

### 4.8. Improvements in Spinal Literacy

The overall improvement in health literacy suggests that the intervention empowered students and provided them with tools to manage modifiable risk factors, in line with the findings of Mantilla-Toloza et al. (2021) [31] and Pani et al. (2024) [11], who report significant gains in postural knowledge and self-care competencies following educational programs. However, the lack of behavioral change in stair-climbing practices supports the conclusions of Minghelli et al. (2021) [19]: sustainable postural habits require interventions that incorporate active practice, individualized activities, and sustained repetition, systematically embedded in the school curriculum to facilitate long-term automatization.

### 4.9. Limitations

This study has several limitations that should be considered when interpreting the results. First, the absence of a control group limits the ability to attribute the observed changes exclusively to the intervention, as improvements may have been influenced by external or uncontrolled factors. Therefore, causal inferences should be made with caution. Although sex, age, school, school grade and baseline pain intensity were not analytically controlled and may have influenced the observed outcomes.

Second, the study relied on an ad hoc questionnaire that has not been previously validated. Although the instrument was specifically designed to address the objectives of the intervention, the lack of formal validation may affect the reliability and validity of the measured outcomes. Future studies should consider using standardized and validated assessment tools to strengthen the robustness and comparability of the findings.

## 5. Conclusions

This study suggests that a physiotherapist-led, classroom-based health education program appears to be effective in improving schoolchildren’s postural knowledge and several modifiable behaviors related to spinal health. The intervention was associated with reductions in cervical and thoracic pain and seemed to promote healthier habits, particularly in sitting posture, sleeping position, and backpack load management strategies. These findings indicate that integrating theoretical content with practical demonstrations may be valuable for influencing high-exposure daily behaviors within the school environment.

In contrast, no significant changes were observed in lumbar pain, motor routines requiring automatization (such as rising from bed or carrying backpacks on stairs), or behaviors strongly influenced by the home context, including physical activity levels and screen use. These findings suggest that more complex motor patterns and family-dependent habits may require longer, more intensive, or supervised interventions, as well as increased parental involvement.

Overall, the results support the role of schools as key settings for spinal health promotion and primary prevention. The implementation of standardized, multidimensional, and active educational programs from an early age may contribute to reducing future spinal pain burden and fostering healthier lifestyle behaviors among children.

These findings should be interpreted with caution due to the absence of a control group, the lack of analytical control for potential confounding variables, and the use of a non-validated ad hoc questionnaire.

## Figures and Tables

**Table 1:** Descriptive Characteristics of the Study Variables.

Variable	Pre-Intervention (n; %)	Post-Intervention (n; %)
What sleeping position do they use?		
Supine	50 (15.8%)	42 (13.2%)
Prone	39 (12.3%)	19 (6%)
Side-lying	211 (66.6%)	235 (74.1%)
What posture do they use to get out of bed?		
Lying supine and then standing up	94 (29.7%)	76 (24%)
Turning onto one side, sitting up, and then standing	205 (64.7%)	214 (67.5%)
What posture was adopted when sitting?		
Sitting posture adopted	184 (58%)	213 (67.2%)
Postural strategy used when sitting	74 (23.3%)	43 (13.6%)
Posture assumed during sitting	40 (12.6%)	34 (10.7%)
Sitting posture		
Feet supported on the floor	196 (61.8%)	219 (69.1%)
Feet dangling	62 (19.6%)	37 (11.7%)
Sitting with weight supported on one leg	32 (10.01%)	28 (8.8%)
Sitting on the knees	9 (2.8%)	11 (3.5%)
Type of backpack used		
Backpack worn on the back	244 (77%)	277 (71.6%)
Rolling backpack	56 (17.7%)	53 (16.7%)
How do they place the books inside the backpack?		
Heaviest books placed far from the back	115 (36.3%)	81 (25.6%)
Heaviest books placed close to the back	182 (57.4%)	190 (59.9%)
How do they carry the backpack on the stairs?		
On the back	218 (68.8%)	225 (71%)
With the trolley handle over the shoulder	34 (10.7%)	31 (9.8%)
Carrying the backpack by one hand/holding it by the handle	47 (14.8%)	33 (10.4%)
At what time of day do they check their backpack?		
Every morning	69 (21.8%)	65 (20.5%)
Every night	201 (63.4%)	194 (61.2%)
Never	29 (9.1%)	35 (11%)
How do they believe the weight should be arranged inside the backpack?		
Heaviest items placed away from the ribs	104 (32.8%)	72 (27.7%)
Heaviest items placed close to the ribs	151 (47.6%)	189 (59.6%)
It makes no difference	45 (14.2%)	19 (6%)
How do they believe the backpack should be carried when going up the stairs?		
On the back with both shoulder straps centered	222 (70%)	245 (77.3%)
With the trolley handle or backpack handle placed over one shoulder	29 (9.1%)	18 (5.7%)
Holding the backpack by the handle with one hand	49 (15.5%)	28 (8.8%)
Which sleeping position do they believe is correct?		
Supine	104 (32.8%)	94 (29.7%)
Prone	24 (7.6%)	8 (2.5%)
Side-lying	171 (53.9%)	192 (60.6%)
Which method do they believe is the correct way to get out of bed?		
Transitioning from a supine position directly to standing	89 (28.1%)	56 (17.7%)
Rolling onto one side, sitting up, and then standing	211 (66.6%)	237 (74.8%)

## Data Availability

Data are contained within the article. The data are not publicly available due to participants’ privacy.

## References

[1] Fraiz-Barbeito M., Rey-Veiga S., González-González Y., Da Cuña-Carrera I., Alonso-Calvete A., Santamaría Solís M.T. (2021). Epidemiología del dolor raquídeo en una población de escolares de España. Arch. Argent. Pediatr..

[2] Louw Q., Kriel R.I., Brink Y., van Niekerk S.M., Tawa N. (2021). Perspectives of spinal health within the school setting in a South African rural region: A qualitative study. Work.

[3] Szilágyi B., Tardi P., Magyar B., Tanács-Gulyás N., Romhányi F., Vida E., Makai A., Járomi M. (2021). Health questionnaire on back care knowledge and spine disease prevention for 6–10 years old children: Development and psychometric evaluation. BMC Musculoskelet. Disord..

[4] García-Moreno J.M., Calvo-Muñoz I., Gómez-Conesa A., López-López J.A. (2024). Assessment of the Effects of Physiotherapy on Back Care and Prevention of Non-Specific Low Back Pain in Children and Adolescents: A Systematic Review and Meta-Analysis. Healthcare.

[5] Noll M., Candotti C.T., da Rosa B.N., Vieira A., Loss J.F. (2021). Back pain and its risk factors in Brazilian adolescents: A longitudinal study. Br. J. Pain.

[6] Calvo-Muñoz I., Kovacs F.M., Roqué M., Seco-Calvo J. (2020). The association between the weight of schoolbags and low back pain among schoolchildren: A systematic review, meta-analysis and individual patient data meta-analysis. Eur. J. Pain.

[7] Galmes-Panades A.M., Borràs P.A., Vidal-Conti J. (2023). Association of postural education and postural hygiene with low back pain in schoolchildren: Cross-sectional results from the PEPE study. Health Promot. Perspect..

[8] Miño C., Gutiérrez-Espinoza H., Olivares-Arancibia J., Yáñez-Sepúlveda R., Duclos-Bastías D., Araya-Quintanilla F., Smith L., López-Gil J.F. (2025). Prevalence of Chronic Back Pain and Associated Factors in Children and Adolescents: Secondary Analysis of the 2001–2019 Health Behavior in School-Aged Children Study. JMIR Public Health Surveill..

[9] Oka G.A., Ranade A.S., Shinde M.K., Pore P.D. (2025). Screen time reduction and Surya Namaskars—A comprehensive intervention for nonspecific back pain in adolescents: A study protocol. Front. Pediatr..

[10] Alibegović A., Mačak Hadžiomerović A., Pašalić A., Domljan D. (2020). School Furniture Ergonomics in Prevention of Pupils’ Poor Sitting Posture. Drv. Ind..

[11] Pani S.M., Gaccetta F., Cadoni F., Della Salda A., Liori A., Contu P. (2024). Pilot evaluation of the effectiveness of an ergonomics awareness educational programme addressed to middle-school children. Glob. Health Promot..

[12] Anyachukwu C.C., Amarah C.C., Atueyi B.C., Anthony I., Nweke M., Abaraogu U. (2024). Effectiveness of Back care education Programme among school children: A systematic review of randomized controlled trials. BMC Pediatr..

[13] Fazaa A., Cherif I., Miladi S., Boussaa H., Makhlouf Y., Abdelghani K.B., Laatar A. (2024). Prevalence of spine pain among Tunisian children and adolescents and related factors. Pediatr. Rheumatol..

[14] Tereshchenko S., Kasparov E., Manchuk V., Evert L., Zaitseva O., Smolnikova M., Shubina M., Gorbacheva N., Novitckii I., Moskalenko O. (2024). Recurrent pain symptoms among adolescents with generalized and specific problematic internet use: A large-scale cross-sectional study. Comput. Hum. Behav. Rep..

[15] Li C., Zhao Y., Yu Z., Han X., Lin X., Wen L. (2022). Sagittal imbalance of the spine is associated with poor sitting posture among primary and secondary school students in China: A cross-sectional study. BMC Musculoskelet. Disord..

[16] Vidal J., Borràs P.A., Ponseti F.J., Cantallops J., Ortega F.B., Palou P. (2013). Effects of a postural education program on school backpack habits related to low back pain in children. Eur. Spine J..

[17] Park J., Kim J.S. (2011). Effects of spinal health educational programs for elementary school children. J. Spec. Pediatr. Nurs..

[18] Szilágyi B. (2022). Examination and Development of Back care Knowledge and Spine Disease Prevention Among 6-10 Years Old Children. Ph.D. Thesis.

[19] Minghelli B., Nunes C., Oliveira R. (2021). Back School Postural Education Program: Comparison of Two Types of Interventions in Improving Ergonomic Knowledge about Postures and Reducing Low Back Pain in Adolescents. Int. J. Environ. Res. Public Health.

[20] Heiser S.L. (2014). Effects of a Back Pain Prevention Education Program on Knowledge of Proper Back Care Among Fifth Grade Elementary Students. Doctoral Dissertation.

[21] Fiaschi Ramos A.C., Ferreira Silva R.M., Bizinotto T., Teixeira de Rezende L.M., Miñana-Signes V., Monfort-Pañego M., Noll P.R., Noll M. (2022). Tools for Assessing Knowledge of Back Health in Adolescents: A Systematic Review Protocol. Healthcare.

[22] Ozdemir S., Gencbas D., Tosun B., Bebis H., Sinan O. (2021). Musculoskeletal Pain, Related Factors, and Posture Profiles Among Adolescents: A Cross-Sectional Study from Turkey. Pain Manag. Nurs..

[23] De Moura Alves M.E., Alves J.M.S.R.O., Moreira C.A.R., Ferreira L.F.B.M., Soares C.M.S., Flores P.M.T., Forte P.M.G., Miradouro J.C.S. (2022). Cross-cultural validation of a questionnaire to assess spinal pain in children. Rev. Bras. Ortop..

[24] Foltran F.A., Moreira R.F., Komatsu M.O., Falconi M.F., Sato T.O. (2012). Effects of an educational back care program on Brazilian schoolchildren’s knowledge regarding back pain prevention. Braz. J. Phys. Ther..

[25] Cruz del Moral R., Zagalaz-Sánchez M.L., Molero D., Cachón-Zagalaz J. (2016). Validación de un cuestionario para la cuantificación del dolor de espalda en escolares. Rev. Cuba. Salud Pública.

[26] Chambers C.T., Dol J., Tutelman P.R., Langley C.L., Parker J.A., Cormier B.T., Macfarlane G.J., Jones G.T., Chapman D., Proudfoot N. (2024). The prevalence of chronic pain in children and adolescents: A systematic review update and meta-analysis. Pain.

[27] López-Hernández T., Caparó-Ferré M., Giné-Martí S., Salvat-Salvat I. (2020). Relationship between School Backpacks and Musculoskeletal Pain in Children 8 to 10 Years of Age: An Observational, Cross-Sectional and Analytical Study. Int. J. Environ. Res. Public Health.

[28] Akbari-Chehrehbargh Z., Tavafian S.S., Montazeri A. (2020). Effectiveness of a theory-based back care intervention on spine-related behavior among pupils: A school-based randomised controlled trial (T-Bak study). BMC Public Health.

[29] Ludwig-Walz H., Siemens W., Heinisch S., Dannheim I., Loss J., Bujard M. (2023). How the COVID-19 pandemic and related school closures reduce physical activity among children and adolescents in the WHO European Region: A systematic review and meta-analysis. Int. J. Behav. Nutr. Phys. Act..

[30] Basuodan R.M., Gmmash A., Alghadier M., Albesher R.A. (2024). Relationship between Pain, Physical Activity, Screen Time and Age among Young Children during the COVID-19 Pandemic. Healthcare.

[31] Mantilla Toloza S.C., Jaimes Guerrero C.A., Lerma Castaño P.R. (2021). Knowledge and Practices of Back Care, Experience in Colombian Children. Glob. Pediatr. Health.

